# Effectiveness of Advanced Practice Nursing Interventions on Diabetic Patients: A Systematic Review

**DOI:** 10.3390/healthcare13070738

**Published:** 2025-03-26

**Authors:** Ana Rodríguez-García, Álvaro Borrallo-Riego, Eleonora Magni, María Dolores Guerra-Martín

**Affiliations:** 1 Andalusian Health Service, Virgen Macarena Hospital, 41009 Seville, Spain; anarg1992@gmail.com; 2 Nursing Department, Faculty of Nursing, Physiotherapy and Podiatry, University of Seville, 41009 Seville, Spain; guema@us.es; 3 Institute of Biomedicine of Seville (IBiS), 41013 Seville, Spain

**Keywords:** advanced practice nursing, nurse practitioners, clinical nurses, practice patterns, nurses, diabetes mellitus

## Abstract

**Background:** Diabetes mellitus is a complex chronic condition requiring continuous healthcare. Consequently, various organisations recommend therapeutic education to enhance treatment adherence. This is often facilitated by Advanced Practice Nurses, who provide a range of advanced interventions that impact clinical health outcomes and deliver healthcare services to these patients. **Objective:** To analyse the effectiveness of interventions performed by Advanced Practice Nurses in patients with diabetes. **Method:** A peer-reviewed systematic review was conducted and registered in PROSPERO. The databases consulted included PubMed, Scopus, Web of Science, and CINAHL. Inclusion criteria comprised studies published between 2014 and 2024 on the effectiveness of interventions by Advanced Practice Nurses in diabetic patients. The review included qualitative, quantitative, and mixed methods designs. Various screenings were carried out, including the assessment of methodological quality. **Results:** A total of 600 studies were identified, of which 17 were selected for final review. Among these, 12 studies focused on diabetic education. Interventions were predominantly delivered in person in primary care settings, private clinics, and hospitals. Reported outcomes included reductions in HbA1c levels, improved patient self-knowledge and self-efficacy, and decreased rates of readmission and mortality. **Conclusions:** The sample consisted predominantly of women over 60 years of age. Diabetic education emerged as the most common intervention, primarily delivered in person by Advanced Practice Nurses across diverse settings. Nearly all interventions proved effective in improving health outcomes for diabetic patients.

## 1. Introduction

Throughout time, Nursing has progressively evolved in the entire world, with the emergence of the Advanced Practice Nurse (APN) figure, which was defined by the International Nursing Council as a nurse who, through additional training, has acquired expert knowledge, complex decision-making skills, and clinical competences to expand their practice. All this exerts a direct influence on clinical health outcomes and on the provision of direct services to individuals, families, and communities. The characteristics of an APN can vary according to the context of the country where they develop their activities, as variations can be noticed in health systems, in the regulatory mechanisms for this figure, and in nursing training systems [1].

It is to be taken into account that the figure of APN was born in the United States at the beginning of the 20th century in the form of two roles: Clinical Nurse Specialists (CNSs) and Nurse Practitioners (NPs). Clinical Nurse Specialists usually concentrate in more indirect care, more linked to non-clinical activities such as supporting the system, education, leadership, and research, from a systemic approach more connected to in-hospital environments [1,2,3]. Nurse Practitioners concentrate more on direct care, more on clinical activities such as diagnosis, treatment, and prescriptions for the patients in different clinical environments; they are more autonomous professionals and are linked to Primary Health Care [1,2,4,5]. Nevertheless, there are other terms identified with APNs, such as expert nurses, midwifery nurses, nurse anaesthetists, outpatient care nurses, emergency nurses, and liaison nurses or case-management nurses, among others [5,6,7,8].

Following the United States, the figure of the APN was developed in Canada and in other countries such as the United Kingdom, Australia, New Zealand, Netherlands, Sweden, and Ireland. It did not reach Spain until the beginning of the 21st century, when there was a change in the organizational models to adapt health services to the population’s needs. This change was implemented in the form of different figures across different health services, mostly as Case-Management Nurse [9,10]. In fact, in Spain, this figure poses a major political, legislative, and educational challenge, with the mandatory requirement to devise laws that regulate it, define its profile, and regulate its process and integration into the Spanish health system, as well as to develop ruled university teaching strategies that favour sound development of clinical skills, knowledge, and vision to perform its duties [2].

With its varied denominations, the APN is an important figure in terms of support for generalist nurses due to the difficulties they face in their everyday practice, as the patients’ conditions are increasingly complex and demanding, which requires high care quality standards [9]. It is of utmost importance to highlight that the care provided to chronic patients constitutes one of the main challenges for most health systems [10].

In some cases, living with chronic disease can turn out to be difficult, even more so when the required and self-administered treatment and control are complex. In fact, therapeutic non-adherence is one of the main problems for chronicity care to be effective. In this regard, the APN plays a fundamental role in improving this care [11].

Several organizations speak about incorporating an APN profile for the diabetes approach, in order to promote complex case resolution and active education in the patients [11,12]. Diabetes Mellitus (DM) is a complex chronic disease that affects people of all ages and social statuses and requires continuing health care in charge of a multidisciplinary team, with multi-factorial strategies for risk reduction beyond glycaemic control. Various international consensuses recommend resorting to therapeutic education in DM by means of team comprising expert professionals with guaranteed knowledge; this is crucial for achieving good results in the patients, as it will help promote quality of life improvements, better treatment adherence, and reductions in the number of complications [11].

Based on the above, the objective proposed is to analyse the effectiveness of interventions carried out by Advanced Practice Nurses in patients with diabetes.

## 2. Materials and Methods

### 2.1. Study Design

A peer systematic review was carried out, following the Preferred Reporting Items for Systematic Reviews and Meta-Analyses (PRISMA) statement and the Cochrane manual for intervention systematic reviews, which ensure the conduction of complete and rigorous reviews [13,14]. The study protocol was registered in PROSPERO (CRD42023407829) before selection and data extraction.

### 2.2. Search Strategies and Selection Criteria

The following databases were consulted to identify relevant studies: PubMed, Web of Science, Scopus, and CINAHL. Two reviewers conducted the searches independently from the keywords identified on the theme. The search strategy was: (“Nurse Practitioner” OR “Clinical Nurse Specialist” OR “APN” OR “Advanced Practice Nursing” OR “Practice Patterns, Nurses’”) AND “Diabetes Mellitus” NOT “Review”.

The study selection criteria were as follows: (1) published studies on the effectiveness of advanced practice nurse interventions in patients with diabetes; (2) studies employing qualitative, quantitative, and mixed methods designs published between 2014 and 2024. The studies (including protocols and projects) in which outcomes of interest were neither measured nor reported were not eligible.

### 2.3. Data Analysis and Assessment of Article Quality

Two reviewers selected the studies independently according to their titles and abstracts and, subsequently, as per the pre-established eligibility criteria. All disagreements regarding study selection were resolved by consensus with a third author. Subsequently, two reviewers separately extracted the data from the selected articles, and all authors participated in the discussion and synthesis of the results. A registration form was designed following the indications set forth in the Cochrane Manual [14], detailing (a) authorship and year; (b) study design, period, and country; (c) the ages and sexes of participants, as well as whether they belonged to a control group (CG) or an intervention group (IG), if specified in the study design. Additionally, the outcomes measured in each study, the instruments used for measurement (where applicable), the interventions performed by the APN, and their effectiveness were recorded.

The methodological quality of the included studies was assessed by the two independent reviewers using the following instruments (in case of disagreement, a third author was consulted):

1. In qualitative studies, those scoring below 50% on the *Standards for Reporting Qualitative Research* (SRQR) were excluded. This instrument consists of 21 items divided into five dimensions, providing a framework and recommendations for reporting this type of research. The following categorization, based on the percentage of items meeting the evaluation criteria, was applied: Excellent (80–100%), Good (50–80%), Fair (30–50%), and Poor (<30%) [15];
2. In observational studies, those scoring below 12 on the *Strengthening the Reporting of Observational Studies in Epidemiology* (STROBE) checklist were excluded. This instrument consists of 22 items, with a maximum possible score of 22. Studies with scores <12 were deemed to be of insufficient quality [16];
3. In Randomised Controlled Trials (RCTs), studies scoring below 4 on the *Physiotherapy Evidence Database* (PEDro) scale were excluded. This instrument comprises 11 items, each with a binary response (“Yes” or “No”), where each affirmative response scores 1 point. The maximum score is 10, as the first item is not considered due to its relevance to the external validity of the studies. The methodological quality was categorized as follows: Excellent (9–10 points), Good (8–6 points), Acceptable (5–4 points), and Poor (<4 points). Studies scoring below 4 were of insufficient quality [17,18,19];
4. In mixed-method studies, those scoring below 4 on the *Good Reporting of a Mixed Methods Study* (GRAMMS) scale were excluded. This instrument consists of six items, with a maximum possible score of 6. Studies scoring <4 were classified as being of insufficient quality [16,20];
5. In quasi-experimental studies, those scoring below 12 on the *Transparent Reporting of Evaluations with Nonrandomised Designs* (TREND) checklist were excluded. This instrument comprises 22 items grouped into five domains: title and abstract, introduction, methodology, results, and discussion [21,22]. The same scoring criteria as the STROBE checklist were applied, given the identical number of items and the lack of evidence for a specific cut-off score. Accordingly, studies with scores <12 were of insufficient quality.

### 2.4. Risk of Bias Analysis

The Cochrane Risk of Bias (ROB-2) tool (V.2) was employed to assess the risk of bias in RCTs [23,24,25], while the JBI critical appraisal tool was utilised for quasi-experimental studies [26,27].

## 3. Results

### 3.1. Presentation of the Studies

**Figure 1:**
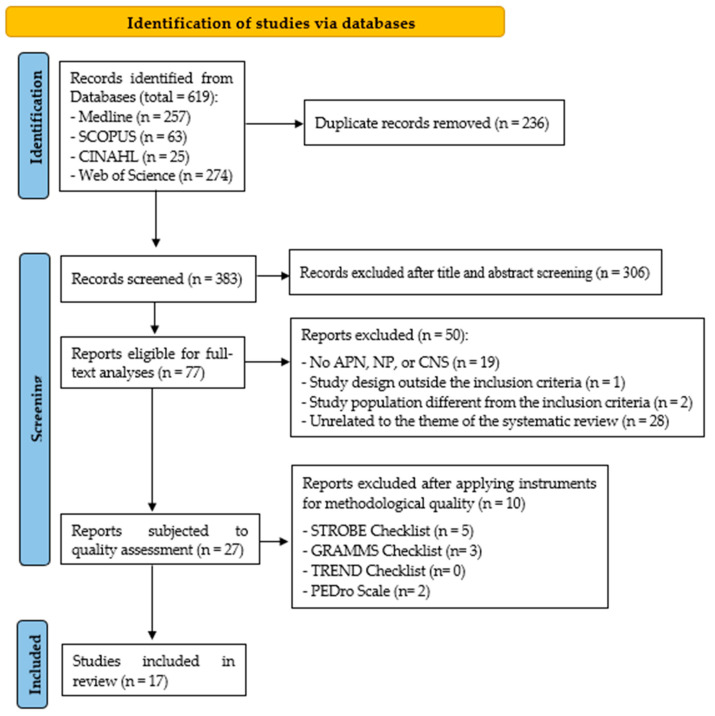
PRISMA flow diagram of study.

A total of seventeen studies met the inclusion criteria and were included (Figure 1). The characteristics of the studies are presented in Table S1.

### 3.2. Quality Assessment and Risk of Bias

Table S2 presents the results obtained in the third screening, following the application of various tools to assess the methodological quality of the 28 studies selected after the second screening. At the end of the third screening, 17 studies were finally selected, as depicted in the flow chart.

Table S3 and Figure S1 display the results of applying the Cochrane Risk of Bias (ROB-2) tool for RCTs. Table S4 presents the results of using the JBI critical appraisal tool for quasi-experimental studies.

### 3.3. Thematic Analysis

#### 3.3.1. Characteristics of the Diabetic Patients Subjected to APN Interventions

Table 1 shows the different characteristics of the sample comprising diabetic patients subjected to APN interventions. The samples of the studies consisted both of women and of men [28,29,30,31,32,33,34,35,36,37,38,39,40,41,42,43]. In 50% of them, there was a higher percentage of women in the sample [28,29,31,33,34,36,39,41]; 43.75% had a higher percentage of men [32,35,37,38,40,42,43]; and the percentage of men and women was the same in 6.25% [30]. In relation to gender, one study made no reference to this aspect [44].

As for the diabetic patients’ age, the overall mean found in the studies was 64.5 years old. It is to be noted that 23.53% of the studies had samples comprising people aged between 30 and 50 years old [28,30,34,44], whereas 76.5% included participants aged at least 60 years old [29,31,32,33,35,36,37,38,39,40,41,42,43].

Regarding the DM diagnosis in the samples from the studies, 35.3% of the patients had Type 2 DM (DM2) [32,33,36,39,40,42]. One study differentiated its sample between Type 1 DM (DM1) and DM2 [44]. Another one made a distinction between the patients with DM2 and those without [37]. Nevertheless, the type of diabetes presented by the patients was not specified in 41.18% [29,31,34,35,38,41,43]. In addition, the patients had DM (without specifying its type) along with other chronic diseases in two studies [28,30].

#### 3.3.2. APN Interventions in Diabetic Patients and Their Effectiveness

Table 2 presents the variables and/or instruments used in the studies, as well as the APN interventions carried out and their effectiveness.

As for the interventions, 70.59% of the studies focused on diabetic education [28,29,32,33,34,35,36,37,38,39,43,44]. In 35.29% of them, the interventions were targeted at lifestyle changes, treatment adherence, prevention of complications, and drug prescription [28,33,36,37,39,43]. Diabetic education was implemented in combination with insulin therapy training in two studies [29,32]. Finally, in other cases, diabetic education was combined with telemonitoring support [38] or with insulin self-injection simulations, in turn promoting fewer fears, concerns, and myths [34]. One study combined education with support and motivation-improving measures [35]. Another one included supervision by means of a device called the Abbott FreeStyle Precision Pro and changing the treatment [44]. Two studies make reference to the need to train, supervise, and work as a team with other professionals when it comes to implementing and promoting interventions carried out by APNs [40,41].

Resuming the topic of the interventions themselves, the following findings were observed as for their in-person or remote modality: (1) exclusive in-person modality in 76.47% of the studies [28,29,30,31,32,34,35,36,37,39,40,42,44]; (2) exclusive remote modality in two cases, via videoconferences [43] or phone calls [33]; (3) in-person/remote combined modality in another two studies [38,41]. In the cases with in-person modality, they were carried out in hospital centres in six studies [29,32,35,37,40,44], in Primary Health Care centres in five cases [28,30,34,36,42], and in two in private clinics [31,39].

In relation to the professional figure that was in charge of the interventions, they were carried out independently by APNs in 64.7% of the cases [30,31,32,34,36,37,38,39,42,43,44]; in the other cases (35.3%), the interventions were jointly implemented by an APN along with other health professionals such as physicians, endocrinologists, educators specialized in diabetes, or community health workers [28,29,33,35,40,41].

As for the effectiveness of the APN interventions in diabetic patients, we should make it clear that they were focused on clinical results (HbA1c, LDL, or BP) in 76.47% of the studies [28,29,30,32,33,34,35,36,37,38,39,40,42]. Two of them were RCTs, which allowed comparing the IG results against those obtained in the CG [28,33], finding significant improvements in the IG clinical outcomes in only one of the studies [28]. In the case of the observational studies [29,30,35,36,40,42], it was possible to reduce the patients’ HbA1c levels at the end of the intervention; however, in two studies [29,36], the results were only attained when the intervention was carried out by an APN against the one implemented by another professional. This reduction in the HbA1c levels after the intervention was also described in the quasi-experimental studies [32,34,38,39,41].

In 41.18% of the studies, the interventions were focused on the self-knowledge and self-efficacy attained by the patients after the APN intervention [30,31,34,38,39,41,43]. The patients’ self-knowledge and self-efficacy in managing their pathology were improved in all of them at the end of the intervention, which helped improve treatment adherence and self-care, thus increasing empowerment in diabetes management [30,34,38,39,41,43]. In one of these studies, it is also stated that it is fundamental to establish an adequate patient–APN relationship, as it helps improve the clinical outcomes and reduce the emotional distress sometimes related to diabetes [38]. Despite these findings, a reduction in treatment adherence between the patients cared for by an APN and those served by another professional was observed in another of the studies selected. Nevertheless, the patients cared for by an APN presented fewer comorbidities, complications, and hospitalizations when compared to those served by another professional [31].

In 29.41% of the cases, the hospital admission and mortality rates stood out among their variables, in addition to hospitalization time or costs [28,31,32,37,44]. Two of these studies reported that an intervention led by an APN reduced hospital readmission rates among the treated patients [32,44]. It was also possible to reduce the mortality rate and mean hospitalization time among the patients at the end of the intervention in two of the studies selected [37,44]. Three studies addressed the costs [28,31,37]. In this sense, one of these studies stated that the cost of an intervention in charge of an APN was similar to that of one carried out by another professional [31]. However, another study indicated that the intervention carried by the APN had a higher cost when compared to the other control intervention [28].

#### 3.3.3. Improvement Strategies in Relation to APNs

Improvement strategies were described in 12 studies [28,29,30,31,32,34,36,39,41,42,43,44]; they are presented in Table 3.

## 4. Discussion

### 4.1. Characteristics of the Diabetic Patients Subjected to APN Interventions

In the studies selected, some differences regarding the characteristics of the samples can be noticed. In relation to the diabetic patients’ age, the samples in most of the studies presented mean ages over 60 years old [29,31,32,33,35,36,37,38,39,40,41,42,44]. This finding is in line with other authors, whose studies show mean ages that are similar to the ones described by the studies included in the current review [45,46]. It is to be noted that DM2 is linked to aging in many cases, with its prevalence increasing with age, which can intensify many of the geriatric symptoms [47,48].

As for gender, most the studies had a predominance of women in their samples [28,29,31,33,34,36,39,41]. Various authors have incorporated higher percentages of women in their samples [46]. Nevertheless, other studies with samples comprising the same number of men and women have been conducted [45]. It is also important to consider that diabetes affects each gender in a different way, both in terms of its clinical repercussion and of its impact [49,50]. It is for this reason that it is important to consider the existing biases in health care between men and women, as many of the current social determinants cause late diabetes recognition and delays in care and treatment initiation among women, which leads to deficient control of the pathology [49].

### 4.2. APN Interventions in Diabetic Patients and Their Effectiveness

Various studies have highlighted the challenges that may arise in diabetes management, such as recurrent hypoglycaemia or persistent hyperglycaemia. In this regard, APN-led interventions focused on diabetes education are key to improving diabetes management and preventing complications. In fact, it was the intervention most commonly led by APNs and cited in the majority of the reviewed studies [28,29,32,33,34,35,36,37,38,39,43,44]. Various studies have also incorporated this intervention, both to enhance patients’ knowledge and self-efficacy [51] and to improve their biochemical parameters [52]. Other authors support this intervention by promoting the inclusion of complementary group dynamics that improve the interaction among individuals with the same pathology. This, in turn, fosters experience sharing, clarifies doubts, facilitates the acquisition of new knowledge, and reduces the impact of the pathology on a person’s quality of life [53].

Another of the interventions proposed is especially linked to working as a multidisciplinary team, where APNs should foster their training and supervision [40,41]. In fact, some authors stress the importance for APNs to work in an interdisciplinary way, following structured coordination of the care provided in order to improve and ensure the patients’ quality of life [54]. This aspect is also crucial to improve comprehensive care [31,55].

As for the modality of the interventions (in-person, remote, or mixed), it was in-person in most of the studies selected [28,29,30,31,32,34,35,36,37,39,40,42,44]. This coincides with various studies already conducted on the theme [39,45,52,56]. Other studies included the remote modality by resorting to videoconferences or phone calls. Some authors have put forward the suitability of incorporating interventions through mobile apps or other remote devices [57]. The mixed modality has also been described in studies from the current review [38,41], which finds a number of authors who mention the usefulness of incorporating the in-person modality of intervention along with remote ones, such as phone calls [57].

In relation to the professional figure that was in charge of the interventions, they were carried out independently by APNs in most of the studies [30,31,32,34,36,37,38,39,42,43,44], as already described in other studies alien to this review [45,55]. In other studies included, the APNs worked along with other professionals in implementing the interventions [28,29,33,35,40,41]. These findings are in line with other authors who stress interdisciplinary work coordinated with other professionals to improve the care provided and ensure patient care quality [54].

Regarding the effectiveness of interventions led by APNs, it should be considered that nearly all studies have designed their interventions to subsequently analyse changes in patients’ clinical outcomes, especially those focused on the HbA1c levels. In general, it was possible to reduce these levels in almost all the studies [28,29,30,32,34,35,36,38,39,40,41,42], approaching the recommended values. This coincides with the postulates set forth by various authors who, when developing APN therapeutic education programs with patients on insulin, observed a reduction in the HbA1c levels [45].

Another of the aspects assessed in the studies selected was determining how the interventions influenced the self-knowledge and self-efficacy levels attained by the patients. In this case, they were improved and increased in a large part of the studies [30,34,38,39,41,43]. This is consistent with other authors, who indicate that these aspects also lead to an improvement in treatment adherence and, in many cases, to insulin injections, if necessary [45].

The readmission rates, hospitalisation time, mortality rate, and costs were equally addressed in some of the studies selected, showing reductions in general [28,31,32,37,44]. In this sense, other authors have stated how APN interventions in a hospital emergency service have allowed reducing the readmission rates, as well as the patients’ hospitalisation times [55]. In the study conducted by Ordoñez-Piedra et al. [58], it was possible to reduce the readmission and mortality rates, as well as the costs. In relation to the costs, various authors indicate that coordination between APNs and case-management nurses not only improves the care provided to the patients and eases their access to these health services but also helps reduces the economic impact on the consumption of hospital supplies and the costs associated with non-efficiencies. APNs manage to reduce healthcare costs by being well prepared to address the challenges of care focused on health promotion and prevention actions, while also standardising health education [59]. Regarding cost-effectiveness, some authors indicate that APNs are generally cost-efficient providers. However, as their role, scope of practice, and payment mechanisms vary by country, further research with clearly defined cost measures is needed to better understand the potential of APNs to reduce the high cost of healthcare services [60].

### 4.3. Improvement Strategies in Relation to APNs

Some of the studies selected indicate the need to increase the number of APN professionals as an improvement strategy regarding the care provided to diabetic patients, as these professionals help improve the health outcomes [28,31,32,36]. This finding coincides with the postulates set forth by other authors, who highlight this professional figure because it has specialised knowledge and skills that help provide effective and good quality care [61]. To increase the number of APNs and achieve greater integration in diabetes management, it is necessary to convince key decision-makers of their value, promote educational programmes for their training, and improve regulatory frameworks and policies [62,63].

The following have also been described as improvement strategies: APNs incorporating innovative methods to improve the results, such as performing self-injection simulations, glucose continuous monitoring, or incorporating new technologies like videoconference sessions [30,34,42,43]. Various authors have also made reference to the suitability of introducing new methods, including resorting to telehealth options for health care, which helped improve diabetes self-control [64]. Nevertheless, when referring to telehealth as an alternative care method during the COVID-19 pandemic, worsened diabetes quality measures were observed in another study already carried out [65].

Another of the improvement strategies proposed in the studies was to foster diabetic education during hospitalisation, not limiting it exclusively to primary care services. This will foster better treatment adherence after the patients’ discharge [39,44]. In another study, it is described how the care provided by APNs during hospitalisation improved treatment adherence and increased the knowledge levels. This helped reduce the HbA1c levels, the hospitalization times, and the number of readmissions after discharge [66].

The following is also proposed among the improvement strategies: training and qualifying other professionals in diabetes management to support the specialised team and increase profitability [29,41]. This coincides with other authors alien to the review, who mention how training and qualification of other professionals by APNs (such as health support workers) helps reduce APNs’ workload and improve the results obtained [67].

### 4.4. Limitations

The present study has certain limitations, which are as follows: 1. Heterogeneity in the design and methodology of the selected studies. However, several authors consider this aspect a source of valuable information for research when conducted methodically [14]. 2. Regarding the quality and validity of the studies, it should be noted that the majority were either observational or quasi-experimental in design, which entails a higher risk of bias compared to the RCTs included in the review. In this regard, it should also be considered that for quasi-experimental studies, due to the lack of evidence to establish a cut-off point for distinguishing between good or poor quality, the same reference threshold was applied as for purely observational studies, using the STROBE checklist. 3. Restricting the search strategy to the last 10 years.

## 5. Conclusions

Regarding the characteristics of the sample, most of the studies included patients diagnosed with DM2, over the age of 60, and with a higher percentage of women.

As for the interventions carried out by APNs, significant diversity was observed in terms of intervention type, as well as in their number, length in time, modality, and the professional figure in charge of the sessions conducted. The intervention most frequently described was diabetic education, exclusively implemented by APNs in most of the cases, and in the in-person modality, both in primary care environments and in in-hospital ones. Referring to effectiveness, the most effective interventions are those focused on therapeutic education, in which APNs coordinate with the rest of the healthcare team and families. This requires a high level of training to address the challenges of diabetes management. The APN interventions were able to improve the clinical outcomes in general, especially those related to the HbA1c levels. Likewise, the patients’ self-knowledge and self-efficacy in diabetes management are improved, which favours treatment adherence and reducing possible future complications. In addition, it has been described how APN interventions in these patients helps reduce readmission and mortality rates, hospitalisation times, and costs.

In relation to the improvement strategies, it becomes necessary to increase the number of APNs in the different health scopes to enhance care quality and the patients’ quality of life. Likewise, promoting diabetic education (both in primary care and in in-hospital settings), fostering the introduction of new care methods, and encouraging training and qualification of other professionals in diabetes management as support for APNs.

## Figures and Tables

**Table 1:** Characteristics of the samples of diabetic patients with intervention by the APN.

Authors	Sample (Age and Sex)
Allen et al. [28]	A total of 525 patients diagnosed with CVD, DM2, HT, and hypercholesterolemia. IG: 261 patients—187 women (71.7%) and 74 men. Mean age: 54 ± 12 years. CG: 264 patients—187 women (70.8%) and 77 men. Mean age: 55 ± 11.5 years.
Mackey et al. [29]	A total of 714 hospitalised diabetic patients. IG: 171 diabetic patients with 222 hospitalisations—108 women (63.15%) and 63 men. Mean age: 61 ± 13 years. CG: 543 diabetic patients with 665 hospitalisations—483 women (88.95%) and 60 men. Mean age: 68 ± 13 years.
Richardson et al. [30]	26 patients with DM2, with a mean age of 58 years. The sample consisted of 13 women and 13 men. Only one patient had DM2 alone, while the remaining 25 combined DM2 with another chronic condition (HT and/or hyperlipidaemia).
Kuo et al. [31]	A total of 64,354 patients with DM2: Group 1: 14,811 patients, 67.8% of whom were women. The mean age was 76 ± 6.2 years. Group 2: 49,543 patients, 66.1% of whom were women. The mean age was 78 ± 6.5 years.
Brumm et al. [32]	IG: 40 diabetic patients, 95% of whom had DM2. Women accounted for 12.5% (n = 5). The mean age was 64 ± 11.4 years. CG: Patients were compiled from historical aggregated data from the internal patient evaluation centre regarding diabetes-related readmissions in 2014.
Garg et al. [33]	A total of 151 patients with DM2: IG: 77 patients, 58% (n = 45) were women. The mean age was 65 ± 9.4 years. CG: 74 patients, 59% (n = 44) were women. The mean age was 63 ± 10.5 years.
Kuo et al. [34]	A total of 241 diabetic patients: 53.11% (n = 128) were women. The mean age was 56 ± 11 years. The sample was divided into two groups: IG: 162 patients, 53.70% (n = 87) were women. The mean age was 58 ± 11 years. CG: 79 patients, 51.89% (n = 41) were women. The mean age was 52 ± 10 years.
Gardiner et al. [35]	A total of 67 diabetic patients referred at discharge to the hospital’s diabetes service due to a history of uncontrolled diabetes. Women accounted for 47.76% (n = 32). The mean age was 67 years.
Marin et al. [36]	A total of 269 patients with DM2 were divided into two groups: IG: 93 patients, 66.7% (n = 62) were women. The mean age was 62 ± 11.6 years. CG: 176 patients, 65.3% (n = 115) were women. The mean age was 63 ± 10.9 years.
Akiboye et al. [37]	In total, 16,102 patients from the pre-intervention study, of whom 2337 had diabetes. Among these, 43.33% (n = 1106) were women. The mean age was 71 years. At 6 months post-intervention, 17,353 patients were assessed, of whom 2433 had diabetes. Among these, 45.5% (n = 1107) were women. The mean age was 71 years.
Knee et al. [44]	A total of 979 diabetic patients were divided into two groups: Pre-intervention group: 443 patients with a mean age of 59 years. Of these, 46.3% had DM1, 48.5% had DM2, and 5.2% had another type of diabetes. Post-intervention group: 536 patients with a mean age of 62 years. Of these, 40.1% had DM1, 53.5% had DM2, and 6.3% had another type of diabetes.
McGloin et al. [38]	A total of 40 diabetic patients: 42.5% (n = 17) were women. The mean age was 62 years.
Kulsick et al. [39]	A total of 39 patients with diabetes: Pre-intervention: 23 patients, 78.26% were women. Age ranged from 65 to >85 years. Post-intervention: 16 patients, 68.75% were women. Age ranged from 65 to >85 years.
Yago-Esteban et al. [40]	Diabetic patients: Phase 1: 101 patients, 30% of whom were women. The mean age was 71 ± 11 years. Phase 2: 685 patients, 29% of whom were women. The mean age was 69 ± 12 years. Phase 3: 73 patients, 22% of whom were women. The mean age was 63 ± 12 years.
Marsh et al. [41]	A total of 16 patients with diabetes: 75% (n = 9) were women. The mean age was 68 ± 3.5 years.
Dimond [42]	81 patients with DM2, 42% (n = 34) of whom were women. The mean age was 76 years.
Ju et al. [43]	A total of 39 diabetic patients, 36% of whom were women. The age range was from 30 to over 60 years, with 51% of patients aged between 50–59 years.

DM: Diabetes Mellitus; CVD: cerebrovascular disease; HT: hypertension; IG: intervention group; CG: control group.

**Table 2:** Variables and/or instruments, interventions carried out by APNs, and their effectiveness.

Authors	Variables and/or Instruments	Interventions	Effectiveness
Allen et al. [28]	HbA1c, BP, and cholesterol. They were measured from the initial evaluation to the follow-up one. The costs were assessed after one year.	Length of the intervention in time: 1 year. IG: An NP and a community health worker were in charge of implementing a program to reduce the BVD risks. The NP carried out evidence-based educational and behavioural interventions for lifestyle changes and treatment adherence, drug prescription, supervision of the other health professionals, and consultations with a physician. Telephone follow-up was implemented between the visits. The other professional was in charge of reinforcing the NP’s indications. CG: a physician provided feedback on the BVD risks.	At 12 months of the intervention, the IG improved significantly in terms of the LDL, AP, and HbA1c clinical outcomes when compared to the Cg. These findings were also observed during the 12-month follow-up. The total cost after one year of intervention was higher in the IG than in the CG.
Mackey et al. [29]	NP–PA relationship in diabetes care and in using basal-bolus insulin therapy (GEE method) The care provided by NPs/PAs was associated to lower glucose values. It was measured in the first and last 24 hospitalization hours. The care provided by NPs/PAs was associated to lower glucose values. It was measured in the first and last 24 hospitalization hours.	Length of the intervention in time: the patients’ hospitalization time. IG: Diabetes control and diabetic education in hospitalized diabetic patients in charge of an NP and an assistant physician, along with an endocrinologist. Each professional welcomes the patient, performs baseline evaluation, and initiates a preliminary treatment plan, which includes insulin therapy with dose adjustments, discharge recommendations about diabetes, and follow-up with an endocrinologist, if necessary. The process was reviewed by an endocrinologist. CG: patients not receiving care from an NP, assistant physician, or endocrinologist, but from another professional.	A larger reduction in the HbA1c levels was observed between the first and last 24 h in the IG patients, when compared to the CG. Basal-bolus insulin therapy was administered to 80% of the IG patients and to 34% of the CG ones. As for diabetes joint management, the IG reduced the mean HbA1c level by 6.96 units in the last 24 h when compared to the CG.
Richardson et al. [30]	HbA1c, BP, cholesterol, and body weight. Depression (PHQ-9) and self-efficacy (DES-SF). Everything was measured at the beginning and end of the intervention.	Length of the intervention in time: not specified. The Healthcare Effectiveness Data and Information Set (HEDIS) primary care tool was used, which measures and evaluates data and information about the efficacy of the health care provided by NPs. As a first step, the patients’ medical histories were reviewed; subsequently, an individualized treatment plan was defined for each patient according to their medical history, clinical data, medications in use, and social factors. Various data were collected form the patients before and after the intervention for comparison purposes. After the intervention, the patients were followed-up every 2–5 weeks for 5 months. In-person and electronic visits were combined with phone calls. The care frequency was based on each patient’s needs. If necessary, other professionals were consulted due to the patients’ complex health situation.	A large part of the NP’s contact with the patients in the interventions was via phone calls. Self-efficacy improved after the intervention. Albeit slightly, the depression scores were reduced. A total of 50% of the patients reached the HbA1c levels defined as target in the study (<8%), as was the case with the BP and cholesterol levels.
Kuo et al. [31]	Number of ophthalmologic exams, cholesterol, HbA1c, and nephropathy monitoring. Care continuity (MMCI).	Length of the intervention in time: not specified. The HEDIS primary care tool was used, which measures and evaluates the set of and information about the efficacy of the health care provided. IG: the HEDIS program was employed to select those patients comprehensively cared for by an APN to assess their undergoing of tests and exams related to cholesterol, retinographies, HbA1c, nephropathy, and treatment adherence. CG: the HEDIS program was employed to select those patients comprehensively cared for by a physician to assess their undergoing of tests and exams related to cholesterol, retinographies, HbA1c, nephropathy, and treatment adherence.	The IG presented fewer comorbidities, DM complications, hospitalizations, and visits to professionals the previous year than the CG. A lower probability of undergoing retinographies or HbA1c tests was observed in the IG. The IG presented less continuing care and more visits to specialists than the CG. The IG had lower DM treatment adherence than the CG. The expenses were similar in both groups.
Brumm et al. [32]	Rehospitalisation rates at 30 days, measuring them during a one-year period. HbA1c, measured during a 3–8-month period.	Length of the intervention in time: 19 months. IG: in charge of an APN specialised din diabetes; the participants were offered a diabetes transition program where they were instructed about survival skills (prevention, recognition, hypoglycaemia treatment, healthy habits, insulin administration, foot care, etc.). They received face-to-face visits, an information booklet before discharge, and weekly follow-up calls for 30 days. CG: in charge of a PHC nurse; the participants were provided standard care before discharge, providing them with education on diabetes self-control. On certain occasions, the patients received a follow-up call after discharge.	A total of 20% of the patients had in-person visits and none of them was readmitted in the 30 days after discharge. A total of 33 patients had their HbA1c levels collected before and after the intervention These levels were significantly reduced; from 11.3% to 9.1%. A total of 11 patients were not administered insulin; 10 had their HbA1c levels collected before and after the intervention. A reduction in these levels (from 11.6% to 7.8%) was observed in these patients. The readmission rate at 30 days was lower in the IG against the CG.
Garg et al. [33]	HbA1c, measured at baseline, at 3 months, and 1 year after discharge. BMI, BP, lipids, renal function, and urine albumin.	Length of the intervention in time: 1 year. IG: Care provided by an NP specialised in diabetes in collaboration with an endocrinologist, via weekly or monthly phone calls to review the HbA1c levels. Advice on diet, physical exercise, and medications was provided. CG: Follow-up in charge of a PHC physician.	There were no significant differences between the patients discharged with continuing insulin (IG: 3; CG: 8) and without insulin (IG: 4; CG 26) in either of the two groups. There were no significant differences between both groups in terms of HbA1c reduction at 3 months and 1 year after discharge. There was also no association between HbA1c reduction and more successful or total phone calls made by the NP.
Kuo et al. [34]	HbA1c, measured before and after the intervention. Number of people who underwent the self-injection simulation; it was measured after the intervention.	Length of the intervention in time: 1 day. IG: Diabetes program implemented by a hospital where a 2 h group visit was carried out in a given month with group of two–eight patients in charge of three NPs and one CSN specialised in diabetes. The first hour was focused on fears, concerns, myths, erroneous concepts, glycaemic self-control, preventing complications, etc. In turn, the second hour was focused on the practice. After the group visit, the patients could return to their PHC physician, undergo telephone or in-person follow-up with their NP, or attend a follow-up group. CG: Standard care in charge of another professional.	A total of 54.7% of the IG patients initiated insulin treatment, against 39.4% from the CG. 92% of the IG patients were successful in their self-injection simulations. The HbA1c level was reduced by 13.7% in the IG, 15.3% in those who initiated insulin, and 16% in those who performed an injection simulation and started using insulin thereafter. It only presented a 0.56% reduction in the CG during the same time period. By 2–6 months after the intervention, 54.8% of the IG patients managed to reduce their HbA1c levels.
Gardiner et al. [35]	HbA1c, measured at baseline and 3 months after the intervention.	Length of the intervention in time: not specified. Education in diabetes for diabetic patients taught by an NP specialised in the disease and a part-time educator in diabetes. An attempt was made to empower the patients by providing them with knowledge, motivation, and support to help them prevent complications in diabetes.	There were significant differences in the HbA1c levels before and after the intervention: from 13.3 mmol/L (before) to 11.2 mmol/L (after). There was a significant reduction in the HbA1c al levels when comparing the results at the discharge moment to those obtained 3 months after discharge.
Marin et al. [36]	HbA1c was measured at baseline and at 6, 12, and 24 months post-intervention.	Duration of the intervention: not specified.	At baseline, the IG had higher HbA1c levels than the CG, and this difference persisted across the three follow-up points. However, a reduction in HbA1c values was observed in the IG compared to pre-intervention levels. In the CG, HbA1c values remained similar at baseline and across all follow-up points.
		IG: The APN implemented the intervention using the chronic care model. The APN provided patients with counselling and education on lifestyle changes, including physical activity, and discussions on barriers to achieving goals. Prior to the intervention, an assessment of cardiovascular risk factors and physical examinations was conducted.	
		CG: The physician also implemented the intervention using the chronic care model.	Patients who received care from an APN continued with this care over time, whereas some patients initially attended by a physician eventually sought care from an APN.
		After each visit with the APN, a copy of the patient’s progress was sent to the physician.	
Akiboye et al. [37]	Hospitalization time, mortality rate, and readmission rate at 30 days. They were measured 6 months before and after the intervention.	Length of the intervention in time: during hospitalization. Implementation of the DICE program, designed and developed to offer education and care to hospitalised diabetic patients. Each professional is provided with an eight-page booklet to fill in during the process of caring for a diabetic patient. It includes aspects to improve patient safety, control, and instructions for foot exams through a tactile test and how to refer to a multidisciplinary team through the Diabetic Patient At Risk (DPAR) scoring system. The program includes an electronic system to identify diabetic people and another intended for hypoglycaemic alerts.	In the people with DM2, the mortality rate was more significantly reduced after the intervention. Albeit to a slightly lesser extent, it was reduced in those without DM2. The mean hospitalization time was also reduced in both groups after the intervention. Likewise, it was possible to reduce the hospitalization time in the people with DM. This variable was unchanged in the patients without DM. For people with or without DM2, readmission showed a significant increase after the intervention.
Knee et al. [44]	Hospitalization time, readmission rate at 30 days, and mortality rate at 30 days.	Length of the intervention in time: not specified. Installation of a care point system called the Abbott FreeStyle Precision Pro in acute medical emergency rooms highlighting hypoglycaemic, hyperglycaemic, or ketosis values. A control panel on the glucose values was set up. It was managed by a nurse specialised in hospitalised patients with diabetes; she was in charge of reviewing the device (glucose monitoring), intervened in medication exchange, and provided diabetic education and support to patients and professionals in such areas. Pre-I G: patients from 2017. Post-I G: patients from 2018.	There were referrals due to hypoglycaemia in 40.98% of the Pre-I G participants and in 34.8% of the Post-I G ones. As for the patients’ readmissions at 30 days, there was a reduction in the Post-I G, with 20.1% of all the patients against 29.3% of all the Pre-I G patients. The mortality rate at 30 days was also lower in the Post-I G, with 10.8% against 11.5% in the Pre-I Group. The readmission rate fell from 29.9% in the Pre-I G to 20.1% in the Post-I Group. The readmission rate was also reduced in the patients not treated with insulin in the Pre-I G, with 28.1% against 20.4% in the Post-I G. In ICU and DHU patients, the readmission rate fell from 26.6% in the Pre-I Group to 21.4% in the Post-I G.
McGloin et al. [38]	HbA1c and BMI were measured at baseline and 6 and 12 months after the intervention. Self-efficacy (DES), distress (DDS), and satisfaction with the intervention (telemedicine usefulness and satisfaction questionnaire) were measured 6 and 12 months after the intervention.	Length of the intervention in time: 12 weeks. Education in diabetes and diabetic care in charge of CSNs in relation to the Fold TeleCare support, which compared blood glucose readings and insulin level adjustments. In addition, the patients received phone calls and visited the diabetes clinic if necessary.	HbA1c was reduced 6 and 12 months after the follow-up when compared to the baseline values. No changes were recorded in weight or BMI during the six follow-up months. From baseline to 6 months of follow-up, it was possible to enhance empowerment on diabetes and to reduce emotional distress due to diabetes. The mean score for satisfaction with the intervention was above 4 points out of 5. The patients stated greater awareness about self-controlling their disease. The good relationship between patients and CSNs led to increased empowerment in the patients and to better results.
Kulsick et al. [39]	Medication adherence rate, BP, cholesterol, and HbA1c, before and after the intervention.	Length of the intervention in time: 1 year. A number of NPs carried out several diabetic education strategies with the patients attending their office to improve drug management, prescribing consistency, and the NP–patient relationship, in addition to medication reminders. The visits lasted between 30 and 60 min.	The treatment adherence rate (taking the medication as prescribed at least 80% of the time) rose from 85.7% (before) to 94.6% (after). The patients with DM had 80% adherence before the intervention, which improved to 87% after it. In the 13 weeks after the intervention, the HbA1c levels fell from 6.9 mg/dL (before) to 6.7 mg/dL (after), representing a scarcely significant difference.
Yago-Esteban et al. [40]	Hospitalisation time, pharmacological and non-pharmacological treatment types, capillary glycaemia, and HbA1c were measured. An overall satisfaction ad hoc survey was the instrument used to know the professionals’ opinions about the program and the APN role.	Length of the intervention in time: approximately 3 years.	Phase 1: 48% of the patients were prescribed basal-bolus insulin in the first 48 h after admission, to later increase it up to 57%.
		Care and educational therapy standardised program structured in three phases during hospitalisation in the cardiovascular wing, in charge of an APN specialised in diabetes and cardiovascular risk. - Phase 1: observation of the patients admitted, training of the professionals on the insulin calculator for good basal-bolus insulin prescription and habitual practice in patients admitted with high HbA1c levels; - Phase 2: implementation of the program where the physicians used the calculators and the APN worked with the physicians and patients; - Phase 3: follow-up of the patients after discharge by an APN (2 weeks, 4–6 weeks and 6–8 weeks) for those with customised interventions in terms of insulin therapy variation or adaptation. The patients were subsequently referred to PHC or to an endocrinologist.	Phase 2: The APN intervened in 11% of the patients, where 84% had HbA1c > 8%, 55% were in treatment with non-insulin anti-diabetic drugs, and 30% was administered at least two insulin doses. At discharge, the treatment was intensified in 48% of the patients and 36% was administered insulin therapy. Phase 3: The HbA1c levels in the patients identified in Phase 1 decreased after discharge; however, they remained at high levels (from 9.9% to 8.6%). In those that took part in the program, it was possible to reduce HbA1c to adequate levels (from 9.2% to 7.3%). This trend remained unchanged at 3, 6, and 12 months. The blood glucose level was higher in those that were prescribed insulin with the calculator.
Marsh et al. [41]	HbA1c. Diabetes self-care (SDSCA), knowledge (DKQ), and satisfaction level in the patients and health professionals (questionnaire from rural pilot program).	Length of the intervention in time: 12 weeks. COAH-TEAM diabetes program carried out by a community health worker along with other professionals (an NP and a nurse specialised in diabetes, among them). Home visits lasting 1 h were made every 2 weeks for 12 weeks. A routine evaluation and diabetic education were implemented in these visits. Videoconferences with patients and other health professionals were also carried out. The NP and NSD were in charge of training the CHW, in addition to supervising the interventions.	The HbA1c level was significantly reduced from 9.2% to 7.2% after the intervention. As for the diabetes self-care activities, there was an improvement when comparing those at baseline to the ones performed after the intervention. This improvement was also observed in the knowledge level. Regarding satisfaction with the care received, the patients stated being very satisfied with the program. A total of 91% were satisfied with the communication with the care team and the intervention carried out by the CHW.
Dimond [42]	HbA1c and hypoglycaemia onset were measured before and after their detection use.	Length of the intervention in time: 2 years and 6 months. Continued glucose monitoring by placing a sensor due to hyperglycaemia or to high HbA1c levels, hypoglycaemic events, or other reasons. There were two types: (1) Abbot FreeStyle Libre Pro for 14 days in the dorsal side and upper part of the arm; and (2) Dexcom G6 Pro for 10 days in the abdomen. Selection of one or the other depended on the patient and the physician.	There was a reduction in HbA1c in all the cases after incorporating GCM. Likewise, those with at least two GCMs in use noticed a reduction in HbA1c. Hypoglycaemia cases were observed in 48% of the subjects that had previously been applied GCMs due to hyperglycaemic events.
Ju et al. [43]	Feasibility of the program (DKS), summary of diabetes self-care activities (SDSCA), and diabetic foot self-care DFSBS). Usability of the program (telehealth usability questionnaire). They were measured at baseline and at 1.5 and 3 months after the intervention.	Length of the intervention in time: 3 months. Education on diabetic food taught by nurse specialists and guided by the Integrated Theory of Health Behaviour Change. Knowledge on diabetes was taught and information about self-care and care behaviours for the feet was provided. It consisted in one visit per month during 3 months through videoconferences lasting 1 h each. First visit: foot care practice. Second visit: healthy eating habits and maintaining actions with the support from family members. Third visit: reviewing previous concepts and exams to undergo in the consultations to detect neuropathy and peripheral vascular disease.	The participants reported a positive attitude towards using telehealth, with a score of 6.24 out of 7. Knowledge about diabetes, self-care ability, and foot care improved 3 months after the intervention. Likewise, there was increase in the foot exam frequency and the general behaviours for foot care.

BVD: Brain Vascular Disease; NP: Nurse Practitioner; IG: Intervention Group; CG: Control/comparison Group; AP: Arterial Pressure; HbA1c: Glycated haemoglobin; LDL: Low-density cholesterol; BP: Blood Pressure; DM: Diabetes Mellitus; PHC: Primary Health Care; APN: Advance Practice Nurse; CSN: Clinical Specialty Nurse; DM2: Type 2 Diabetes Mellitus; ICU: Intensive Care Unit; DHU: Diabetes Hospital Unit; BMI: Body Mass Index; NSD: Nurse Specialised in Diabetes; GCM: Glucose Continuous Monitoring; GEE: Generalized Estimation Equation; PHQ-9: Patient Health Questionnaire-9; DES-SF: Diabetes Empowerment Scale-Short Form; MMCI: Modified, Modified Continuity Index; DICE: Diabetes Hospitality Care and Education; DDS: Diabetes Distress Scale; Pre-I G: Pre-intervention group; PosI-IG: Post-intervention group; SDSCA: Summary of Diabetes Self-Care Activities measure; DKQ: Diabetes Knowledge Questionnaire; DFSBS: Diabetes Foot Self-are Behaviour Scale; CHW: Community Health Worker.

**Table 3:** Improvement strategies in relation to APNs.

Authors	Improvement Strategy
Allen et al. [28]	Larger incorporation of NPs and community health workers in community health centres, as they help improve care quality.
Mackey et al. [29]	Training other professionals such as NPs in diabetes management through education and managing activities. This way, it will be possible to support the individual services for hospitalised patients and to improve profitability by reducing the work team size.
Richardson et al. [30]	Larger incorporation of innovative methods such as support for the care provided by NPs. This will help improve HbA1c levels, self-efficacy, and the costs associated with visits to the office and with treatment adherence.
Kuo et al. [31]	More care time provided by NPs in consultations and visits. This is a requirement for the care of patients with diabetes, as it will assist in the clinical outcomes and improve prescription safety.
Brumm et al. [32]	Improving the trusting relationship between patients and professionals for better diabetes self-control and health outcomes.
Garg et al. [33]	Not described.
Kuo et al. [34]	Increasing the implementation of insulin therapies in patients with DM2 through group visits in charge of an APN. This will allow reducing psychological barriers regarding insulin use.
Gardiner et al. [35]	Not described.
Marin et al. [36]	Greater use of the APN-Physician model to provide support to the patient. This allows improving the patients’ health outcomes, as it makes use of the APN’s high qualifications, incorporating medical care in more complex cases.
Akiboye et al. [37]	Not described.
Knee et al. [44]	Improving diabetic education during hospitalisation. This ensures a safe and effective hospital–home transition for hospitalised patients.
McGloin et al. [38]	Not described.
Kulsick et al. [39]	Improving the patients’ treatment adherence, chronic disease management, and quality of life through interventions driven by teams not only from Primary Health Care but also from private clinics and hospitals.
Yago-Esteban et al. [40]	Not described.
Marsh et al. [41]	APNs improving the formal training of community health workers in motivational interviews to maximise the impact of self-care behaviours in the patients. In addition, improving virtual care throughout the assistance process.
Dimond [42]	The NPs that work in primary care on diabetes management should consider using glucose continuous monitoring in their practice.
Ju et al. [43]	Improving attendance to videoconferences by creating a website or mobile app so that the patients can be sent reminders or manage their visits. In addition, creating an educational program that incorporates telehealth videoconferences to improve diabetic foot care.

## Data Availability

No additional data are available.

## Supplementary Materials

The following supporting information can be downloaded at: https://www.mdpi.com/article/10.3390/healthcare13070738/s1, Table S1: Study Characteristics; Table S2: Results obtained from assessing the methodological quality of the studies; Table S3: Results of applying the Cochrane Risk of Bias (ROB-2) tool for RCTs; Table S4: Results of using the JBI critical appraisal tool for quasi-experimental studies; Figure S1: Results of applying the Cochrane Risk of Bias (ROB-2) tool for RCTs.
