# Supplementary material for: Effectiveness of Advanced Practice Nursing Interventions on Diabetic Patients: A Systematic Review
Source: Healthcare (Basel). 2025 Mar 26;13(7):738. doi: 10.3390/healthcare13070738 (PMC11989214; doi:10.3390/healthcare13070738)
Supplement: Supplementary file 1 [file healthcare-13-00738-s001.zip › healthcare-3486896-supplementary.pdf]

Table S1. Study Characteristics

Authors	Design/Study Period/Country
Allen et al. [28]	RCT. Conducted between July 2006 and July 2009 in two community health centres in the United States
Mackey et al. [29]	Observational study. Conducted from July to December 2011 in a hospital that is part of an academic tertiary care medical centre in the United States.
Richardson et al. [30]	Observational study. Conducted in 2013 in primary care clinics in the United States.
Kuo et al. [31]	Observational study. Conducted in 2009 in Medicare and Medicaid service centres in the United States.
Brumm et al. [32]	Quasi-experimental study. Conducted from July 2013 to February 2015 in an urban hospital at the Cincinnati VA Medical Centre in the United States.
Garg et al. [33]	RCT. Conducted at Brigham and Women's Hospital (BWH) in the United States.
Kuo et al. [34]	Quasi-experimental study. Conducted between November 2008 and June 2012 in 25 primary care clinics in the United States.
Gardiner et al. [35]	Observational study. Conducted from February 2015 to January 2016 at Calvary University Hospital in Australia.
Marin et al. [36]	Observational study. Conducted between 2007 and 2010 in a chronic preventive care clinic at an outpatient centre in the United States.
Akiboye et al. [37]	Observational study. Conducted between January 2013 and June 2013 at Ipswich Hospital NHS Trust in the United Kingdom.
Knee et al. [38]	Quasi-experimental study. Conducted in two periods: February to July 2017 and February to July 2018 at the Royal Derby Hospital in the United Kingdom.
McGloin et al. [39]	Observational study. Conducted between April 2016 and June 2017 in community clinics in Ireland.
Kulsick et al. [40]	Quasi-experimental study. Conducted in 2019 in several clinics in Amherst, United States.
Yago-Esteban et al. [41]	Observational study. Conducted between October 2017 and February 2019 in a clinical hospital in Barcelona, Spain.
Marsh et al. [42]	Quasi-experimental study. Conducted from September to November 2021 in a federally qualified health centre in the United States.
Dimond [43]	Observational study. Conducted in 2019 in a primary care office in the United States.
Ju et al. [44]	Quasi-experimental study. Conducted in two family medicine clinics in the United States.

RCT: Randomised controlled trial

Table S2. Results obtained from assessing the methodological quality of the studies

Tool	Authors	Score obtained	Excluded
STROBE Checklist	MacKey et al. [29]	13/22	
	Richardson et al. [30]	14/22	
	Kuo et al. [31]	16/22	
	Melaku-Abbera et al. [65]	9/22	X
	Gardiner et al. [35]	14/22	
	Marin et al. [36]	16/22	
	Nelson et al. [66]	9/22	X
	Wright et al. [67]	10/22	X
	Akiboye et al. [37]	15/22	
	Roschkov et al. [68]	10/22	X
	Wright et al. [69]	11/22	X
	Yago-Esteban et al. [41]	14/22	
	Dimond [43]	14/22	
GRAMMS Checklist	Furler et al. [70]	1	X
	Wilkinson et al. [71]	3	X
	Tokuda et al. [72]	3	X
	McGloing et al. [39]	5	
TREND Checklist	Brumm et al. [32]	13	
	Kuo et al. [34]	17	
	Knee et al. [38]	13	
	Kulsik et al. [40]	16	
	Marsh et al. [42]	16	
	Ju et al. [44]	18	
PEDro Scale	Allen et al. [28]	7	
	Apsey et al. [73]	3	X
	Garg et al., 2017 [33]	6	
	Momin et al. [74]	2	X

Table S3. Results of applying the Cochrane Risk of Bias (ROB-2) tool for RCTs

ROB-2	Authors	
	**Allen et al. [28]**	**Garg et al. [33]**
**1. Randomization process**		
1.1. Was the allocation sequence random?	**NI**	**NI**
1.2. Was the allocation sequence concealed until participants were enrolled and assigned to interventions?	**NI**	**NI**
1.3. Did baseline differences between intervention groups suggest a problem with the randomization process?	**N**	**N**
**2. Effect of assignment intervention**		
2.1. Were participants aware of their assigned intervention during the trial?	**Y**	**Y**
2.2. Were carers and people delivering the interventions aware of participants' assigned intervention during the trial?	**Y**	**Y**
2.3. Were there deviations from the intended intervention that arose because of the trial context? (If Yes or NI to 2.1 or 2.2)	**NI**	**NI**
2.4. Were these deviations likely to have affected the outcome? (If Yes or NI to 2.3)	**-**	**-**
2.5. Were these deviations from intended intervention balanced between groups? (If Yes or NI to 2.4)	**-**	**-**
2.6. Was an appropriate analysis used to estimate the effect of assignment to intervention?	**N**	**N**
2.7. Was there potential for a substantial impact (on the result) of the failure to analyse participants in the group to which they were randomized? (If No or NI to 2.6)	**N**	**N**
**3. Effect of adhering to intervention**		
3.1. Were data for this outcome available for all, or nearly all, participants randomized?	**Y**	**N**
3.2. Is there evidence that the result was not biased by missing outcome data? (If No or NI to 3.1)	**-**	**N**
3.3. Could missingness in the outcome depend on its true value? (If No or NI to 3.2)	**-**	**N**
3.4. Is it likely that missingness in the outcome depended on its true value? (If Yes or NI to 3.3)	**-**	**-**
**4. Missing outcomes datas**		
4.1. Was the method of measuring the outcome inappropriate?	**N**	**N**
4.2. Could measurement or ascertainment of the outcome have differed between intervention groups?	**N**	**N**
4.3. Were outcome assessors aware of the intervention received by study participants? (If No or NI to 4.1 or 4.2)	**N**	**N**
4.4. Could assessment of the outcome have been influenced by knowledge of intervention received? (If Yes or NI to 4.3)	**-**	**-**
4.5. Is it likely that assessment of the outcome was influenced by knowledge of intervention received? (If Yes or NI to 4.4)	**-**	**-**
**5. Measurement of the outcome**		
5.1. Were the data that produced this result analysed in accordance with a pre-specified analysis plan that was finalized before unblinded outcome data were available for analysis?	**Y**	**Y**
5.2. Is the numerical result being assessed likely to have been selected, on the basis of the results, from multiple eligible outcome measurements (e.g.scales, definitions, time points) within the outcome domain?	**N**	**N**
5.3. Is the numerical result being assessed likely to have been selected, on the basis of the results, from multiple eligible analyses of the data?	**N**	**N**

NI: No information; N: No; Y: Yes

Table S4. Results of using the JBI critical appraisal tool for quasi-experimental studies

JBI items	Authors					
	**Brumm et al. [32]**	**Kuo et al. [34]**	**Knee et al. [38]**	**Kulsik et al. [40]**	**Marsh et al. [42]**	**Ju et al. [44]**
**Internal Validity**						
**Bias related to temporal precedence**						
1. Is it clear in the study what is the "cause" and what is the "effect"?	Y	Y	Y	Y	Y	Y
**Bias related to selection and allocation**						
2. Was there a control group?	Y	Y	N	N	N	N
**Bias related to confounding factors**						
3. Were participants included in any comparisons similar?	N	Y	Y	Y	Y	Y
**Bias related to administration of intervention/exposure**						
4. Were the participants included in any comparisons receiving similar treatment/care, other than the exposure or intervention of interest?	Y	Y	N	N	Y	Y
**Bias related to assessment, detection and measurement of the outcome**						
5. Were there multiple measurements of the outcome, both pre and post the intervention/exposure?						
Outcome 1	N	Y	Y	Y	Y	Y
Outcome 2	Y	-	Y	Y	Y	Y
Outcome 3	-	-	Y	-	-	Y
6. Were the outcomes of participants included in any comparisons measured in the same way?						
Outcome 1	Y	Y	Y	Y	Y	Y
Outcome 2	N	-	Y	Y	Y	Y
Outcome 3	-	-	Y	-	-	Y
7. Were outcomes measured in a reliable way?						
Outcome 1	Y	Y	Y	N	Y	Y
Outcome 2	Y	-	Y	Y	Y	Y
Outcome 3	-	-	Y	-	-	Y
**Bias related to participant retention**						
8. Was follow-up complete and if not, were differences between groups in terms of their follow-up adequately described and analyzed?						
Outcome 1	N	Y	N	N	N	N
Outcome 2	N	-	N	N	N	N
Outcome 3	-	-	N	-	-	N
**Statistical Conclusion Validity**						
9. Was appropriate statistical analysis used?						
Outcome 1	Y	Y	Y	Y	Y	Y
Outcome 2	Y	-	Y	Y	Y	Y
Outcome 3	-	-	Y	-	-	Y

Y: Yes; N: No; -: No outcome

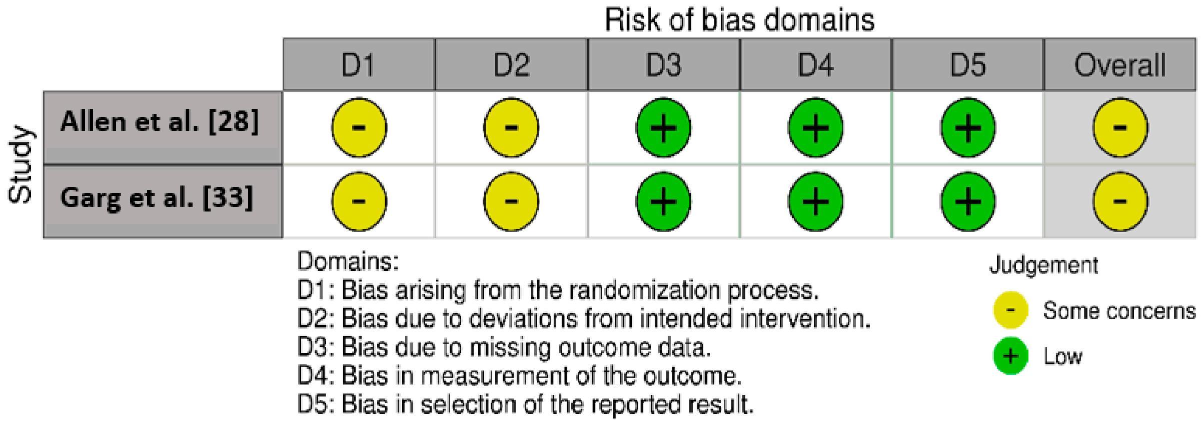

Figure S1. Results of applying the Cochrane Risk of Bias (ROB-2) tool for RCTs. Source: Prepared based on [25]
